# Citrate Supplementation Restores the Impaired Mineralisation Resulting from the Acidic Microenvironment: An In Vitro Study

**DOI:** 10.3390/nu12123779

**Published:** 2020-12-09

**Authors:** Francesca Perut, Gabriela Graziani, Marta Columbaro, Renata Caudarella, Nicola Baldini, Donatella Granchi

**Affiliations:** 1 Biomedical Science and Technology Lab, IRCCS Istituto Ortopedico Rizzoli, via di Barbiano 1/10, 40136 Bologna, Italy; francesca.perut@ior.it (F.P.); nicola.baldini@ior.it (N.B.); 2 Laboratory of Nanobiotechnology, IRCCS Istituto Ortopedico Rizzoli, via di Barbiano 1/10, 40136 Bologna, Italy; gabriela.graziani@ior.it; 3 Electron Microscopy Platform, IRCCS Istituto Ortopedico Rizzoli, via di Barbiano 1/10, 40136 Bologna, Italy; marta.columbaro@ior.it; 4 Maria Cecilia Hospital, GVM Care and Research, Via Corriera 1, 48033 Cotignola (RA), Italy; renata.caudarella@gmail.com; 5 Department of Biomedical and Neuromotor Sciences, Via Pupilli 1, University of Bologna, 40136 Bologna, Italy

**Keywords:** acidosis, bone marrow stromal cells, calcium citrate, potassium citrate, mineralisation, osteoblast, osteoclast

## Abstract

Chronic metabolic acidosis leads to bone-remodelling disorders based on excessive mineral matrix resorption and inhibition of bone formation, but also affects the homeostasis of citrate, which is an essential player in maintaining the acid–base balance and in driving the mineralisation process. This study aimed to investigate the impact of acidosis on the osteogenic properties of bone-forming cells and the effects of citrate supplementation in restoring the osteogenic features impaired by the acidic milieu. For this purpose, human mesenchymal stromal cells were cultured in an osteogenic medium and the extracellular matrix mineralisation was analysed at the micro- and nano-level, both in neutral and acidic conditions and after treatment with calcium citrate and potassium citrate. The acidic milieu significantly decreased the citrate release and hindered the organisation of the extracellular matrix, but the citrate supplementation increased collagen production and, particularly calcium citrate, promoted the mineralisation process. Moreover, the positive effect of citrate supplementation was observed also in the physiological microenvironment. This in vitro study proves that the mineral matrix organisation is influenced by citrate availability in the microenvironment surrounding bone-forming cells, thus providing a biological basis for using citrate-based supplements in the management of bone-remodelling disorders related to chronic low-grade acidosis.

## 1. Introduction

In the past twenty years, a growing body of research has unequivocally demonstrated the strong, causal relationship between acidosis and the pathophysiology of bone disease [1,2,3,4,5,6]. Severe acidosis with acidemia occurs when compensatory measures for maintaining the acid–base equilibrium fail and the blood pH value drops below 7.35, while chronic low-grade acidosis is the result of the continual adaptation of the body to a variety of physiological and pathological conditions, including ageing, menopause, excessive dietary acid intake, bowel diseases, excessive/anaerobic exercise, altered cell metabolism, hypoxia, inflammation, infection, diabetes and tumours [7].

The prompt skeletal response to acute metabolic acidosis is a physicochemical reaction aimed at buffering hydrogen ions by means of alkali metals (sodium, potassium), carbonate and phosphate groups, thus resulting in a net calcium efflux from the mineralised matrix [1,2]. When acidosis follows a more chronic course, it may elicit a cell response. In vitro studies have shown that the bone mineral dissolution due to the ion exchange lasts 48 h on average while, after 48 h, a pivotal role is ascribable to bone cells [2,8]. In fact, the resorption activity of osteoclasts increases dramatically when the intra-bone pH drops below 6.9 and, conversely, acidosis significantly inhibits the osteogenic function of osteoblasts, including the production of extracellular matrix, the activity of alkaline phosphatase and the formation of trabecular bone [4,8].

Contextually to the skeletal modifications, metabolic acidosis has a strong impact on citrate homeostasis. The regulation of the acid–base balance depends largely on acid excretion and urinary buffers, and modulation of the citrate excretion in the kidneys plays a pivotal role in driving this function since proton excess reduces the trivalent anion in the divalent form which may be reabsorbed through the sodium-citrate cotransporter [9]. However, citrate is primarily an intermediate in the tricarboxylic acid cycle (TCA cycle, Krebs cycle), the metabolic pathway which, in humans and all aerobic organisms, is the main adenosine 5′-triphosphate (ATP) provider, i.e., the primary energy source by which living cells accomplish essential functions [10]. When the cellular ATP is abundant and the energy demand of the cells is low, the excess citrate can be exported outside the mitochondria and used for supporting the lipid biosynthesis of proliferating cells [11] or the tissue-related functions of specialised cells, i.e., osteogenic cells [12].

Basically, the pillars of citrate homeostasis are nutritional intake, renal clearance, cellular metabolism and bone remodelling; however, it is currently well known that the main endogenous bulk of citrate is stored in the bone [6,13]. On the one hand, the bone-forming cells are able to synthesise and release citrate into the extracellular matrix so that it may be incorporated into the calcium phosphate–collagen complexes to promote the normal three-dimensional orientation of the apatite nanocrystals in the bone lamellae, thus ensuring the biomechanical properties of bone. On the other hand, the resorption activity by osteoclasts leads to the mobilisation of the citrate incorporated into the mineralised matrix, thus making it the most prominent source for the maintenance of plasma homeostasis [13]. Hence, all the conditions which affect the balance between bone formation and bone resorption potentially affect citrate homeostasis, of which acidosis is part of.

For the above reasons, urinary citrate excretion has been proposed as a laboratory parameter for monitoring the acid–base balance and bone health status, even in subjects without overt metabolic acidosis [14,15,16,17]. Similarly, citrate-based supplements have been used as a possible strategy for treating medical conditions related to acid overload and poor citrate bioavailability, and for mitigating the detrimental effect on skeletal homeostasis. Even though several interventional clinical trials have reported encouraging results, to date, there is no consensus regarding the use of citrate supplementation for the management of metabolic bone diseases since the heterogeneity of the studies did not allow identifying precise indications [6]. Moreover, few studies have dealt with understanding the biological basis of the interaction between extracellular citrate and bone cells, and studies which have investigated the effects under acidic conditions are especially rare. We have recently demonstrated that, independently of its alkalizing capacity, potassium citrate (K citrate) prevented the increase in osteoclast activity induced by the acidic microenvironment while minor effects were observed on bone-forming cells [18]. Other authors have evaluated the activity of citrate under neutral conditions and have demonstrated that increased citrate bioavailability in the extracellular microenvironment may foster the osteogenic differentiation of the mesenchymal stromal cells (MSCs) and accelerate bone tissue regeneration [19,20,21,22,23].

Based on the background, the aim of this study was to investigate the anabolic properties of the citrate-based supplements most widely used in clinical practice, namely calcium citrate (Ca citrate) and K citrate. To attain this result, an experimental in vitro study which allowed evaluating their capability of fostering the osteogenic capacity of human MSCs (hMSCs) in both acidic and neutral settings was designed. First, the effects of acidosis on the osteogenic properties of hMSCs, in particular, on the citrate release and mineralisation of the extracellular matrix was investigated. Then, we evaluated whether the citrate supplementation allowed restoring the impaired functions resulting from exposure to the acidic conditions.

## 2. Materials and Methods

### 2.1. Preparation of Culture Media and Working Solutions of Citrate-Based Compounds

Dulbecco’s Modified Eagle’s Medium (DMEM), Low Glucose-DMEM, and α-minimum essential medium (αMEM) (Sigma-Aldrich, St. Louis, MO, USA) were maintained at pH 6.9 (acidic medium) or 7.4 (neutral medium) by using different concentrations of NaHCO3, according to the Henderson–Hasselbach equation. Complete culture media contained 10% fetal bovine serum (FBS) (Sigma-Aldrich), 20 mM glutamine (Gibco, Monza, Italy), penicillin (100 U/mL) and streptomycin (100 μg/mL) (Thermo Fisher Scientific, Waltham, MA, USA). Osteogenic medium for the mineralisation assay was complete αMEM supplemented with 50 μg/mL-ascorbic acid 2-phosphate, 10^−8^ M dexamethasone, and 10 mM β-glycerophosphate (Sigma-Aldrich). The pH of the culture media was measured before each experiment using a digital pH-meter (6230N, Jenco, San Diego, CA, USA).

The Ca citrate solutions ((C6H5O7)2Ca3*4H2O, Jungbunzlauer Ladenburg GmBH, Germany) were obtained by dissolving the powders in complete αMEM at pH 6.9 or pH 7.4 and stirring overnight at room temperature. The Ca citrate powder solubility reported in the specification sheet was 1.7–17 mM. However, at the higher concentration, the solution of Ca citrate in complete αMEM was cloudy, even after stirring overnight. After subsequent dilution, a clear solution was obtained with Ca citrate 1.5 mM (stock solution).

The stock solution of K citrate-3 mM ((C6H5O7)K3*H2O, Gadot Biochemical Industries Ltd., Haifa Bay, Israel) was obtained by dissolving the powders in complete αMEM at pH 6.9 or pH 7.4, as previously described [18]. The citrate solutions were filtered to avoid contamination of cell cultures. The pH measurement was carried out on samples prepared as for the cell cultures and maintained at 37 °C in a humidified atmosphere of 5% CO_2_.

### 2.2. hMSC Culture and Experimental Plan

**Figure 1:**
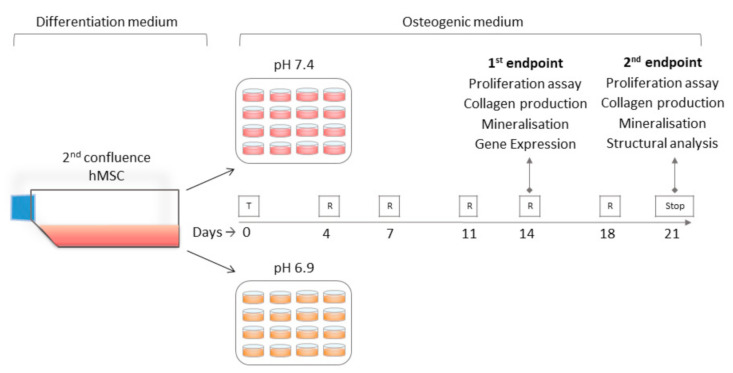
Schematic diagram of the experimental plan. After growing in a differentiation medium, confluent human mesenchymal stromal cells (hMSCs) were cultured with acidic (pH 6.9) and neutral (pH 7.4) osteogenic medium and were treated with four concentrations of two different citrate-based supplements, namely Ca citrate and K citrate (T). The cells were maintained at 37 °C in a humidified atmosphere of 5% CO_2_ and the culture medium was discarded and replaced every 72–96 h (R) until the endpoints which were scheduled at 14 and 21 days were reached (Stop).

The hMSCs were obtained from bone marrow samples collected by reaming the metaphysis and the proximal diaphysis of the femur during reconstructive joint surgery, as previously described [24]. The tissue collection was approved by the internal review board, and informed consent was obtained from the donors. Fifth-passage hMSCs obtained from three male donors (40, 46 and 51 years of age) were maintained in complete DMEM until reaching a second hMSC confluence. The culture medium was then discarded and replaced with acidic (pH 6.9) and neutral (pH 7.4) osteogenic media, and the treatment with Ca citrate and K citrate was started at the same time. The doses were chosen based on the following criteria: (1) lack of toxicity, (2) compensation of the citrate deficit induced by the acidic microenvironment and (3) weight/volume ratio which took into account the chemical formulation of the two compounds ((C6H6O7)2Ca3*4H2O and (C6H5O7)K3*H2O) in order to have comparable citrate concentrations regardless of the source. Figure 1 shows the schematic diagram of the experimental plan.

### 2.3. Alamar Blue Assay

The AlamarBlue^®^ test (Serotec Ltd., Oxford, UK) was used to assess the cellular growth and viability of the hMSC culture following the manufacturer’s recommendations. The cells were seeded onto a 24-well plate (10,000 cell/cm^2^) for 144 h, to evaluate preliminarily the cytotoxicity of citrate-based compounds, and for 14 days and 21 days, as for described in Figure 1. The relative fluorescence unit (RFU) was detected by a microplate fluorescence reader, and the mean of the RFUs measured on two wells was determined. The cell number was extrapolated by using a reference curve constructed by plotting the average RFU of a fixed number of cells in a range from 6000 to 180,000. The correlation between fluorescence emission and cell number was linear (R = 0.94).

### 2.4. Quantification of Citrate Release

The citrate concentration in the supernatant of the hMSC cultures was determined at both endpoints using a colorimetric assay (Citrate Assay Kit, Sigma), following the manufacturer’s recommendations. This method allows detecting citrate concentration in a range of 0.2–10 nmol. Since enzymes in samples could interfere with the assay, the samples were deproteinised with a 10 kDa Molecular Weight Cutoff (MWCO) spin filter (Sartorius, Varedo (ML), Italy) prior to be tested.

### 2.5. Collagen Production

The collagen released from the hMSCs and the collagen deposited into the extracellular matrix were stained by Sirius red dye, as previously described [25]. Briefly, the hMSCs were seeded onto a 24-well plate (5000 cell/cm^2^) and cultured for 14 days and 21 days. The medium was replaced twice a week. The cell supernatant was distributed in 96-well plates (50 µL/well), incubated at 37 °C in a humidified atmosphere for 16 h, and then at 37 °C in a dry atmosphere for 24 h. The wells were then washed three times with double-distilled (dd) H2O 200 μL/well, and 100 µL of 0.1% Sirius red (Polysciences, Inc.; Warrington, PA, UK) in saturated picric acid were added. After 1 h of incubation at room temperature, the wells were washed three times with 10 mM HCl, 200 µL/well, 10 s per wash. The collagen-bound stain was finally eluted with 200 µL/well of 0.1 M NaOH for 5 min and measured using a microplate spectrophotometer at 540 nm. Collagen assessment was carried out in quadruplicate.

Simultaneously, the collagen deposited by the hMSCs was measured. At the endpoints, the cells were washed with phosphate buffered saline (PBS) and fixed in Bouin’s solution for 1 h at room temperature. The samples were washed with ddH2O and stained with 0.1% Sirius red. After washing three times with 10 mM HCl, the collagen deposited by the hMSCs was observed under optical microscopy and, then, the collagen-bound stain was eluted as previously described. Collagen assessment was carried out in duplicate. The presence of Procollagen I N-Terminal Peptide (P1NP) was analysed using an enzyme-linked immunosorbent assay (ELISA) (Cusabio Biotech Co.; Wuhan, China), according to the manufacturer’s protocol.

### 2.6. Quantitative Real-Time Polymerase Chain Reaction (qRT-PCR)

Total RNA was extracted from hMSC cells after 14 days of treatment with Ca citrate and K citrate by using the RNeasy Mini Kit (Qiagen GmbH, Hilden, Germany). Total mRNA was reverse transcribed by the Advantage RT-for-PCR Kit (Roche Diagnostics, Monza, Italy). The expression of “tyrosine 3-monooxygenase/tryptophan 5-monooxygenase activation protein zeta polypeptide” (YWHAZ) (NM_003406), “secreted protein acidic and cysteine rich” (SPARC or osteonectin) (NM_003118.4) and bone sialoprotein (IBSP) (NM_004967.4) were evaluated by qRT-PCR using the Light Cycler instrumentation (Roche Diagnostics). Probes and specific primers were selected using a web-based assay design software (ProbeFinder Software, online available at the Assay Design Center: https://lifescience.roche.com/en_it/brands/universal-probe-library.html#assay-design-center). The sequences of primers selected for the analysis were: YWHAZ-forward 5′-ccgttacttggctgaggttg-3′, YWHAZ-reverse 5′-tgcttgttgtgactgatcgac-3′; SPARC-forward 5′-acccgctttttcgagacc-3′, SPARC-reverse 5′-caagatccttgtcgatatccttct-3′; IBSP-forward 5′-cgaagaaaatggagatgacagtt-3′; IBSP-reverse 5′-cttcattgttttctccttcatttg-3′. The results were expressed as the ratio between gene of interest and YWHAZ, used as reference genes, according to the 2−ΔΔCT method [26].

### 2.7. Mineralisation Assay

To evaluate the mineralisation activity, the hMSC cells were seeded onto a 6-well plate (5000 cell/cm^2^). The mineralising matrix deposition was assessed by Alizarin red dye, as described previously [24], and the calcium nodule formation was examined using microscopy. The stain was then solubilised with a 10% cetylpyridinium chloride solution (p/v) (Sigma-Aldrich), and the optical density was measured at 570 nm. The mineralisation assays were carried out in duplicate.

### 2.8. Transmission Electron Microscopy (TEM) Analysis

Transmission electron microscopy analysis was carried out on the hMSCs (5000 cell/cm^2^) cultured on Thermanox coverslips (Thermo Fisher Scientific, Milano, Italy) for 21 days in neutral and acidic osteogenic medium. At the endpoint, the cell monolayer was fixed with 2.5% glutaraldehyde in 0.1 M cacodylate buffer pH 7.4 for 1 h. After fixation, the samples were post-fixed with 1% osmium tetroxide, dehydrated in a graded series of ethanol, and embedded in Epon. Ultrathin sections were stained with uranyl acetate and lead citrate, and observed with a Jeol Jem-1011 transmission electron microscope (Jeol Jem, Peabody, MA, USA). The images were captured using a Morada digital camera and iTEM software.

### 2.9. Fourier Transform Infrared Spectroscopy (FT-IR) Analysis

The hMSCs (5000 cell/cm^2^) were seeded on Thermanox coverslips (Thermo Fisher Scientific, Milano, Italy) and cultured for 21 days. Before FT-IR acquisition, the samples were rinsed in PBS to eliminate the excess medium. To study the effect of pH on mineralisation, two different methods were used. First, to evaluate the uniformity of mineralisation, the samples were analysed using FT-IR/ATR (attenuated total reflectance) microscopy (Perkin Elmer Spotlight i200, Germanium ATR imaging crystal) using the following parameters: acquisition range 600–4000 cm^−1^, resolution 4 cm^−1^, 16 scans and step size 1 cm^−1^. For each sample, maps were acquired selecting at least 5 non-overlapping 100 × 100 µm^2^ regions in two samples. Then, the cell pellets were detached from the glass substrate and were analysed by FT-IR ATR (Perkin Elmer Spectrum 2, Diamond ATR imaging crystal), using the following parameters: acquisition range 400–4000 cm^−1^, resolution 4 cm^−1^, 32 scans and step size 1 cm^−1^. As a reference for the absence of mineralisation, a curve of the MSCs maintained in differentiation medium for 7 days was acquired. The distribution and organisation of collagen was also compared among different samples, analysing the Amide I and Amide II bands in the 1800–1200 cm^−1^ region.

The effect of citrate-based compounds was evaluated on the cell pellets according to the protocol and parameters described above. For comparison of the extent of mineralisation among the different samples, all the spectra were scaled with respect to the Amide I band at 1644 cm^−1^. A standard curve of non-mineralised MSCs was then subtracted from all the curves. The resulting curves were examined, focusing on the 800–1050 cm^−1^ area to assess the type and relative amount of calcium phosphates formed. To study the differences in collagen organisation, all the samples were scaled with respect to the band at 3277 cm^−1^, and the standard curve of non-mineralised MSCs was subtracted. For treated samples, the reference curve without citrate supplementation, at the same pH value, was also subtracted.

The results were expressed on the basis of a qualitative analysis of the spectra. The type of phase was indicated by specific position of the bands; the variations related to the different culture conditions and treatments were highlighted by comparing the areas underlying the bands, which are proportional to the phase content, as previously shown [27,28].

### 2.10. Calculations and Statistical Analysis

Calculations and statistical analyses were carried out using StatView 5.01 for Windows (SAS Institute Inc., Cary, NC, USA) and MedCalc Statistical Software version 18.2.1 (MedCalc Software bvba, Ostend, Belgium). The quantitative data were expressed as the arithmetic mean plus or minus the standard error of the mean (SEM). The Shapiro–Wilk test was used to test the normality assumption of the continuous variables, and a logarithmic transformation was applied when the data distribution was non-normal. The Pearson correlation coefficient (R) was used to analyse the degree of association between measured parameters. The effects of the acidic milieu and the treatments were analysed by applying the paired t test. Quantitative parameter changes induced by different concentration of citrate supplements were plotted in line charts by calculating the deviations from the baseline using the following formula:

[Treated sample/Control sample] − 1

where the control sample was the culture under standard conditions (osteogenic medium, pH 7.4, citrate 0 mg/mL) and the baseline value was equal to zero. The differences were considered to be statistically significant when the *p* value was <0.05.

## 3. Results

### 3.1. The Acidic Milieu Impairs the Osteogenic Properties of hMSC and the Extracellular Matrix Organisation

**Figure 2:**
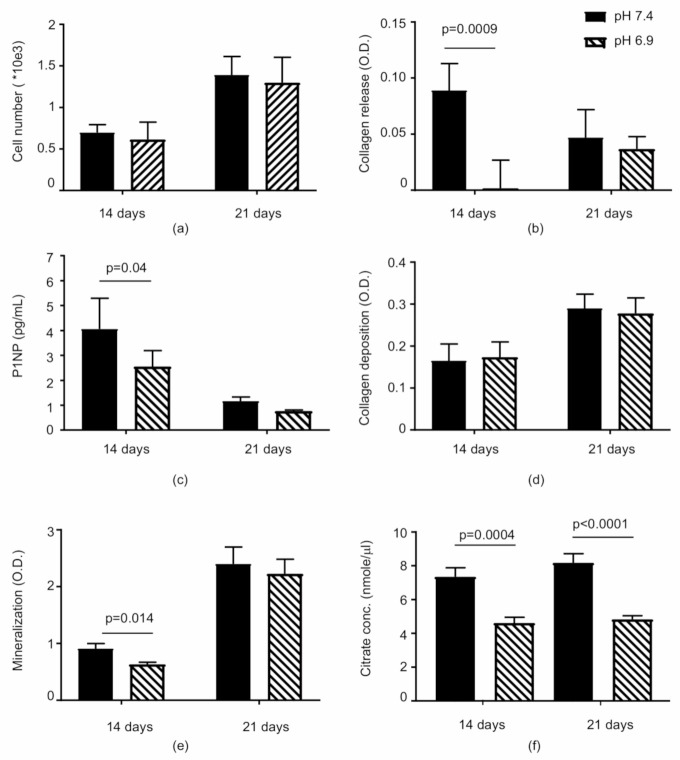
The effects of the acidic milieu on osteoblast functions. The bars represent the mean ± SEM of three separate experiments. The parameters measured after exposure to the osteogenic medium at pH 6.9 and pH 7.4 for both 14 and 21 days were (**a**) the cell number, (**b**) the amount of collagen released in the culture medium (Sirius Red) and (**c**) the amount of procollagen type 1 N-terminal propeptide (PINP), (**d**) the collagen residing in the extracellular matrix (Sirius Red), (**e**) the amount of Alizarin Red bound to the mineralised matrix, and (**f**) the citrate released by the cells.

The most significant effects determined by the acidic microenvironment were observed after 14 days of culture. Cell proliferation was not significantly affected at pH 6.9 as compared to the physiologic pH, and only a slight decrease in cell number was observed (Figure 2a). The amount of collagen lodged into the matrix was identical under both conditions (Figure 2d), but the collagen released from the cells in the culture medium was quantitatively lower after exposure to the acidic milieu (Figure 2b). Moreover, the collagen release was also evaluated by immunoenzymatic assay to detect the amount of procollagen type 1 N-terminal propeptide (PINP) (Figure 2c). The amount of Sirius Red staining correlated significantly with the P1NP (R = 0.54, *p* = 0.0012). A slowing down in the mineralisation process was testified to by the significant decrease in calcium deposition assessed by elution and spectrophotometry measurement of the Alizarin Red S (Figure 2e).

**Figure 3:**
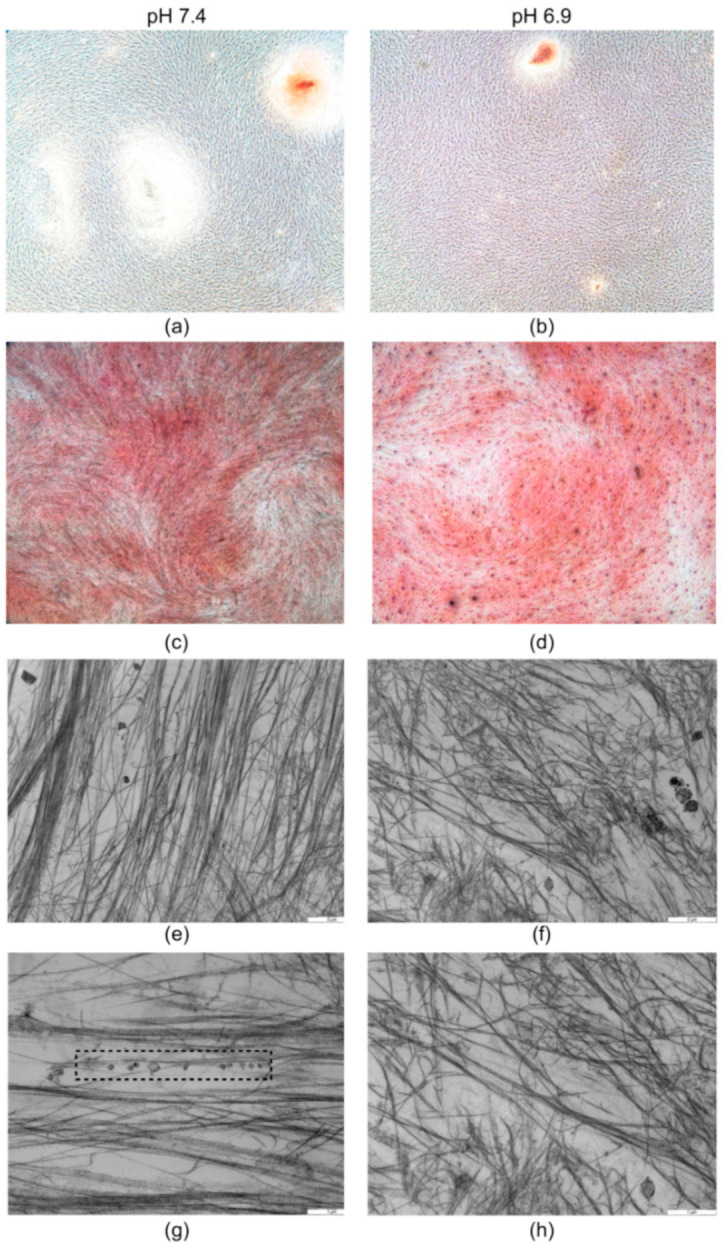
The effects of the acidic milieu on extracellular matrix deposition and organisation. Representative pictures highlight the differences in the organisation of the extracellular matrix formed by the hMSCs after 21 days of culture in neutral (pH 7.4) or acidic (pH 6.9) medium; (**a**,**b**) highlight the differences in number and size of the mineral nodules after exposure to the acidic milieu (Alizarin Red staining, Magnification × 4); (**c**,**d**) representative microscopy images of the collagen in the extracellular matrix (Sirius Red staining, Magnification × 4); (**e**) at pH 7.4, the ultrastructural analysis shows the oriented arrangement of the collagen fibrils bundles; (**g**) numerous non-calcified matrix vesicles are aligned with collagen fibrils in the dashed-line rectangle; (**f**) on the contrary, at pH 6.9, the collagen fibrils are randomly oriented and (**h**) the matrix vesicles are not visible. Bars, (**e**,**f**): 2 μm, (**g**,**h**): 1 μm.

After 21 days of culture at pH 6.9, the cell number and collagen deposited into the extracellular matrix continued to be unaffected (Figure 2d or Figure 3c,d) while the collagen released in the culture medium and calcium deposition increased until reaching an amount detectable at pH 7.4. However, the acidic condition did not allow completing the mineralisation process effectively since a lower number of mineralised nodules, in both the calcified and the under formation phase, were observed (Figure 3a,b). The citrate amount detectable in the culture medium of the hMSCs maintained at pH 6.9 was significantly reduced in comparison to that observed at pH 7.4 (Figure 2f). The mean decrease was approximately 0.57 µg/µL, ranging from −0.516 µg/µL (2.7 nmol/µL) to −0.630 µg/µL (3.3 nmol/µL) measured after 14 and 21 days of culture, respectively.

Additionally, we evaluated the expression of genes involved in the early phase of mineralization process in hMSC cultured for 14 days, i.e., osteonectin/SPARC and IBSP. The analyses were performed in two experiments and presented as supplementary results (Supplementary Figure S1). Under acidic conditions, osteonectin expression decreased significantly (Supplementary Figure S1a), while IBSP expression tended to increase, but not significantly (Supplementary Figure S1b).

Transmission electron microscopy analysis was carried out to examine the extracellular matrix organisation in areas where the mineral nodules were not microscopically visible. The observation of at least ten fields showed clear differences in the organisation of the collagen fibres in the hMSCs cultured at pH 6.9 and pH 7.4. In particular, a disorganised and random distribution was observed in the acidic milieu (Figure 3f,h) while, under neutral conditions, bundles of collagen fibrils appeared oriented but with separate arrangements (Figure 3e,g). Despite the different spatial organisation, the collagen fibres were characterised by a faint periodicity which indicated that the typical internal organisation of the heterotrimers was present under both conditions. There was no evidence of crystalline mineral within the observed areas in either sample. However, numerous matrix vesicles aligned to collagen fibrils were detected at pH 7.4 (Figure 3g) while they were not visible at pH 6.9 (Figure 3h).

**Figure 4:**
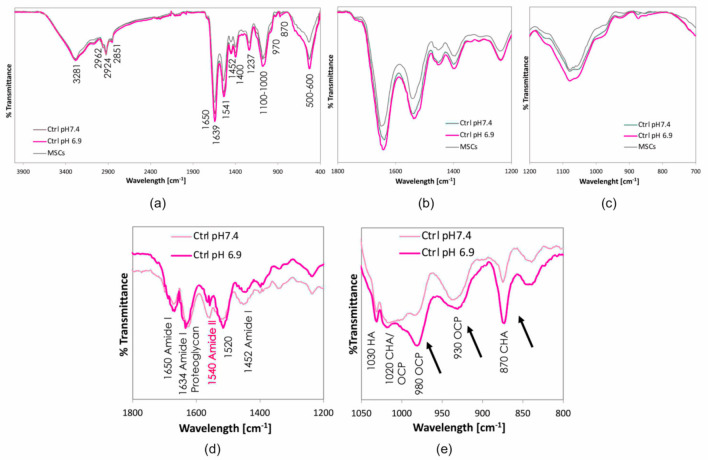
Representative FT-IR spectra of the hMSC samples removed from coverslips and analysed as a whole. The upper panels show the spectra of the cells cultured in the osteogenic medium at pH 6.9 and pH 7.4, and differentiation medium (non-mineralising hMSCs). The position of all bands is reported in (**a**–**c**) highlight the band region corresponding to collagen ad calcium phosphates, respectively. The lower panels show the FT-IR spectra after subtracting the hMSC reference curve; (**d**) bands of collagen and (**e**) bands of calcium phosphates. In (**d**), the Amide I bands under neutral and acidic conditions are overlapping while the intensity of the Amide II band is different. The arrows show a different formation of octacalcium phosphate (band at 980 cm^−1^) with respect to hydroxyapatite and content of carbonates (band at 870 cm^−1^) depending on the acidic or neutral condition. HA: hydroxyapatite; OCP: octacalcium phosphate; CHA: carbonated hydroxyapatite.

To better evaluate the extracellular matrix organisation FT-IR analyses were carried out across all samples in areas where the mineral nodules were not microscopically visible (Supplementary Figure S2a–c). Differences among the spectra were assessed by comparing the position of the bands, which indicated the type of phase that formed and, qualitatively, by the area underlying each band, which was proportional to phase content. Compared to the reference hMSCs, samples at both pH 6.9 and 7.4 showed different profiles, with an increase in the area of the bands in the 1000–1100 cm^−1^ region, corresponding to the spectra of phosphate stretching and, therefore, consistent with the presence of mineralisation activity (Supplementary Figure S2e,f). A high variability was found in the specimens cultured in neutral and acidic osteogenic media. In particular, samples at pH 7.4 showed the formation of distinct bands at ≈1030 cm^−1^, corresponding to the antisymmetric stretching mode of v3PO4 in hydroxyapatite. These bands were present only in part of the samples examined and were absent in other areas, indicating the presence of submicrometric nodules of mineralisation, unevenly distributed in the extracellular matrix (Supplementary Figure S2e). The high variability in the area of the bands at 1000–1100 cm^−1^ did not allow an informative comparison of the effects induced by the two different pHs. For this reason, the spectra were acquired after scraping cells from the substrate and placing the pellet on an ATR detector, thus analysing the composition of each sample as a whole (Figure 4a–e).

All curves showed bands of Amide I, ascribable to the C=O stretching mode of the peptide bond, Amide II, ascribable to the N-H stretching mode of the peptide bond and Amide III, ascribable to the tertiary C-N stretching mode. More specifically, the Amide I bands were detected at 1650 cm^−1^ (ν[C=O]), 1640 cm^−1^ (also compatible with proteoglycans), 1452, and 1400 (δ(CH2) and δ(CH3) absorptions), the Amide II bands were located at ≈1550 cm^−1^ and Amide III was located at 1240 cm^−1^ (C–N stretching and N–H deformation (Figure 4a,b,d). Amide I (1670–1620 cm^−1^) and Amide II (1570–1530 cm^−1^) IR signals are largely recognised as the two main marker bands of collagen; hence, its presence in the samples was confirmed. Bands of calcium phosphates were visible in the 1000–1100 cm^−1^ and 500–600 cm^−1^ areas, deriving from phosphate stretching and bending, respectively. A strong band is also visible at 870 cm^−1^ owing to carbonate groups in carbonated hydroxyapatite (Figure 4a,c,e). To better compare the amount and type of the new calcium phosphates formed, spectra of samples at pH 6.9 and 7.4 were analysed after subtraction of the non-mineralised MSC curves (Figure 4e). Bands characteristic of hydroxyapatite (HA: 1030 cm^−1^), octacalcium phosphate (OCP: 1020 cm^−1^, also characteristic of carbonated hydroxyapatite, 930 cm^−1^) and carbonated hydroxyapatite (CHA: 1020 cm^−1^, 870 cm^−1^ (CO3ν2)) were detected. Spectra at different pHs did not show relevant differences in the amount of newly formed calcium phosphates (extent of mineralisation), as demonstrated by the comparable area underlying the curves. Instead, the mineral phase seemed to be influenced since both the carbonate substitution and the formation of octacalcium phosphate rather than hydroxyapatite were more evident at pH 6.9, as indicated by the area underlying the bands at 870 cm^−1^ and 980 cm^−1^.

As in the case of calcium phosphates, no relevant differences were detected in the amount of collagen formed under neutral and acidic conditions (Figure 4d). However, differences in the intensity of the Amide II band indicated that collagen orientation and organisation could be affected by pH. Instead, the Amide I band, which is related to the collagen secondary structure, was similar in all samples.

### 3.2. Citrate-Based Supplements Affect the Mineralisation Properties of hMSC

Four citrate concentrations were tested starting from the amount which was defective in the culture treated under acidic conditions, namely 0.56, 0.28, 0.14, and 0.07 mg/mL, corresponding to 1.5, 0.75, 0.375, and 0.187 mM of Ca citrate, and 3, 1.5, 0.75, and 0.375 mM of K citrate, respectively. To investigate the alkalizing capability of citrate-based compounds, the supplemented pH 6.9-culture media were maintained at 37 °C in a humidified atmosphere of 5% CO_2_ for 24 h and then the pH value was re-measured. The supplementation was not sufficient to neutralize the acidic medium, and the pH value closer to 7.4 was obtained with K citrate 3 mM (7.27 ± 0.06) (Supplementary Figure S3a). Before proceeding with the experimental plan as shown in Figure 1, a preliminary evaluation of the cytotoxicity of citrate-based compounds was carried out on short-term cultures (144 h). Both compounds did not show a toxic effect and indeed the medium and low doses of Ca citrate promoted cell proliferation (Supplementary Figure S3b–e).

**Figure 5:**
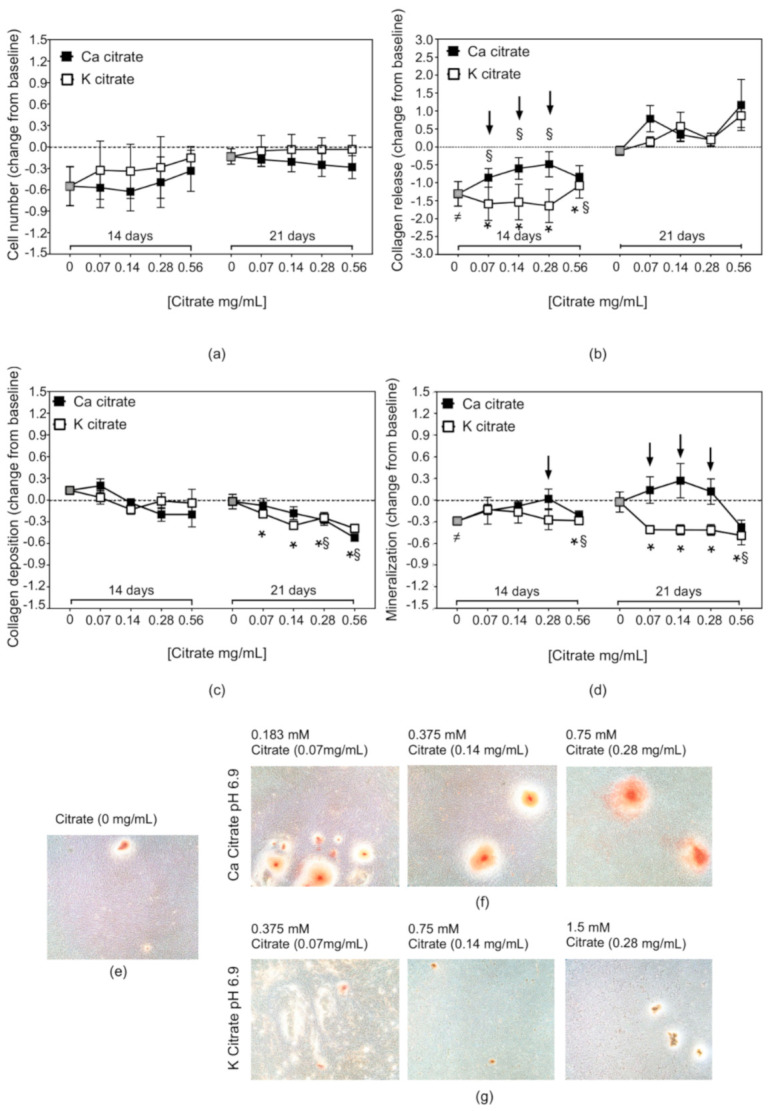
The effects of citrate supplementation on the osteogenic properties of hMSCs cultured under acidic conditions. The line charts represent the mean ± SEM of three separate experiments and show the changes observed after exposure to increasing concentrations of citrate supplements (Ca citrate and K citrate) for 14 and 21 days. The dotted line placed at 0 on the *Y*-axis represents the reference value, that is the result obtained from the hMSC cultures maintained at pH 7.4 without citrate supplementation (control hMSCs). The data are expressed as deviations from the baseline calculated with the following formula: [Treated hMSC/Control hMSC] − 1. The statistical analysis was carried out by comparing the paired samples of each experiment. (**a**) Cell number, (**b**) collagen released in culture medium, (**c**) collagen deposited in the extracellular matrix, and (**d**) mineralised matrix. Microscopy images display (**e**) the effects of the acidic milieu on the matrix mineralisation, the effects of (**f**) Ca citrate and (**g**) K citrate supplementation on the number and the size of mineral nodules (Alizarin Red staining, Magnification × 4). The grey square indicates the result of hMSC cultured at pH 6.9 without supplementation; symbols highlight statistically significant differences (*p* < 0.05) obtained from paired analysis of the data: ^≠^ for untreated hMSC cultured at pH 6.9 versus Control hMSC; * for K citrate-treated hMSC versus Control hMSC; ^§^ for Ca citrate-treated hMSC versus Control hMSC; the arrows for K citrate-treated hMSC versus Ca citrate-treated hMSC.

**Figure 6:**
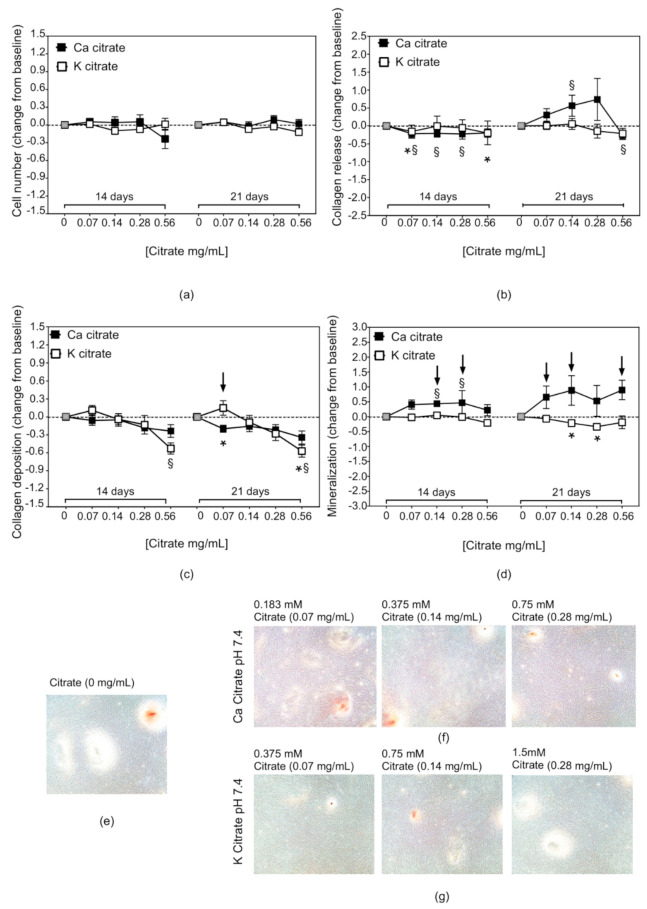
The effects of citrate supplementation on the osteogenic properties of hMSCs cultured under neutral conditions. The results are expressed as in Figure 5. (**a**) Cell number, (**b**) collagen released in culture medium, (**c**) collagen deposited in the extracellular matrix, (**d**) mineralised matrix, and microscopy images displaying (**e**) mineralisation activity in the control cultures and the effects of (**f**) Ca citrate and (**g**) K citrate supplementation on the number and the size of mineral nodules (Alizarin Red staining, Magnification × 4). The grey square indicates the result of hMSC cultured at pH 7.4 without supplementation; symbols highlight statistically significant differences (*p* < 0.05) obtained from paired analysis of the data: * for K citrate-treated hMSC versus Control hMSC, ^§^ for Ca citrate-treated hMSC versus Control hMSC; the arrows for K citrate-treated hMSC versus Ca citrate-treated hMSC.

The effects induced by increasing doses of Ca citrate and K citrate was evaluated under acidic and neutral conditions (Figure 5 and Figure 6, respectively). At pH 6.9, in the first phase of the culture, a slowdown in proliferation with a heterogeneous cell response was observed and the presence of citrate did not change the result significantly. After 21 days, the number of cells was comparable to that found in the control cultures, i.e., hMSCs maintained in the osteogenic medium at pH 7.4 without any supplementation, and no significant differences were observed between the two compounds (Figure 5a).

The remarkable decrease in collagen found in the supernatant of cells cultured under the acidic conditions was still significant after the first 14 days; however, treatment with Ca citrate reduced the differences with the control cultures in a dose-dependent manner, demonstrating a significantly higher activity than K citrate, except for the highest dose 0.56 mg/mL. At 21 days, both compounds induced an increase in collagen bioavailability with mean values higher than those observed in the control cultures (Figure 5b).

In the first phase of the culture, citrate supplementation did not change the amount of collagen residing in the extracellular matrix while, at the second endpoint a significant reduction was observed with all doses of K citrate and with higher concentrations of Ca citrate (Figure 5c).

The citrate-based compounds favoured the recovery of mineralisation which was significantly impaired by the acidic milieu. In fact, after 14 days of supplementation, the amount of Alizarin bound to the matrix did not differ from that measured in the control cultures. Furthermore, Ca citrate affected the mineralisation in a dose-dependent manner and was more active than K citrate, although the higher concentration was found to be ineffective for both compounds. After treatment with citrate-based supplements, we found a highly variable expression of genes involved in the early phase of the mineralization process, and statistically significant changes were undetectable (Supplementary Figure S1c,d). After 21 days, the pro-osteogenic action of Ca citrate was even more evident with mean values higher than those observed in untreated controls, while an opposite effect was observed with K citrate as the deposition of minerals in the extracellular matrix was significantly reduced (Figure 5d–g). The collagen release at 14 days correlated significantly with mineralisation at 21 days (R 0.33, *p* = 0.04) and collagen deposited into the matrix at 21 days (R: 0.578; *p* < 0.0001).

The effects of supplementation were also evaluated at pH 7.4 to explore whether the osteogenic potential of bone-forming cells could be conditioned by the exceeding citrate after the correction of the metabolic defect. The results indicated that Ca citrate had positive effects even under neutral conditions. In fact, the free collagen and the mineralisation were increased after 21 days of treatment as compared to the untreated cultures (Figure 6a–g). Under neutral conditions, the expression of SPARC and IPSP was less variable compared to what observed at pH 6.9. Treatment with citrate-based compounds did not affect SPARC expression, except for a significant downregulation with the higher dose, as observed for collagen deposition. Instead, Ca citrate increased the IBSP expression significantly, in a dose-dependent manner and consistent with data of mineralization at both time points and collagen release at 21 days. At the intermediate doses, Ca citrate demonstrated a significantly higher activity than K citrate (Figures S1e,f).

**Figure 7:**
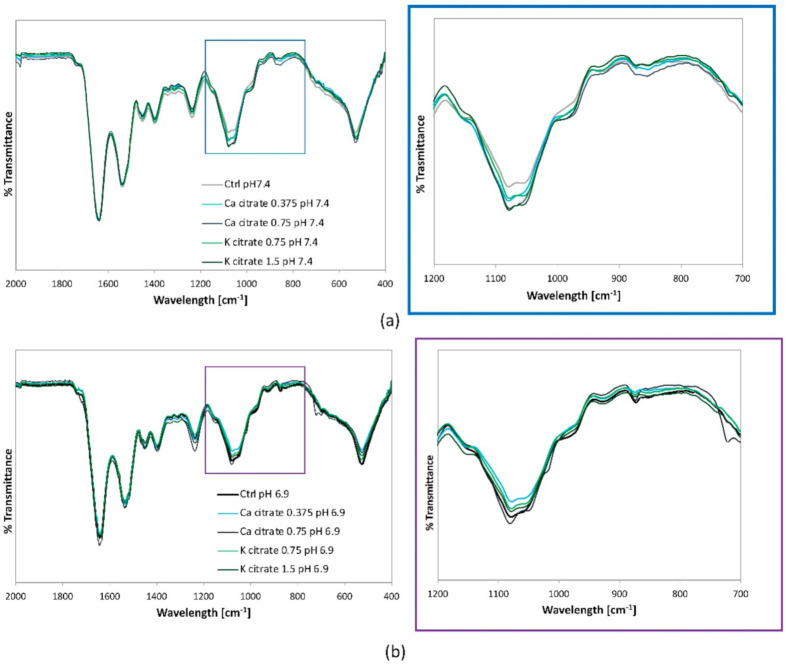
Representative curves of FT-IR spectra of the hMSC samples treated with Ca citrate and K citrate at (**a**) pH 7.4 and (**b**) pH 6.9. Ctrl: hMSC control cultures (pH 7.4, without citrate supplementation).

The effects of the citrate-based supplements on mineralisation activity were also qualitatively evaluated using FT-IR analysis (Figure 7). At pH 7.4, an increase in the intensity of the phosphate stretching band was observed for both Ca citrate and K citrate and the effect correlated with the citrate concentration (Figure 7a). In fact, the mineralisation extent increased with 0.18 mg/mL citrate supplementation (corresponding to Ca citrate 0.375 mM and K citrate 0.75 mM), and even more with 0.36 mg/mL (corresponding to Ca citrate 0.75 mM and K citrate 1.5 mM). In general, the spectra were similar for both compounds, even if the supplementation with K citrate seemed relatively better than that with Ca citrate (Supplementary Figure S4). After subtracting the reference curve without citrate supplementation (ctrl pH 7.4), a shift was visible in the position of the phosphates stretching bands in the 1000–1100 cm^−1^ area, thus suggesting a difference in phase formation (Supplementary Figure S4). The broad and ill-defined shape of the resulting curves, deriving from differences in mineralisation in the presence of citrate very close to the detectability threshold of the instrument, did not allow a univocal attribution of the phases. In the acidic microenvironment, the changes induced by citrate supplementation were not estimable as the variations in submicrometric mineralisation were below the detectability threshold of the instrument (Figure 7b).

## 4. Discussion

Under physiological conditions, the range of pH values measured in the intra-bone blood samples fluctuates from 7.3 to 7.4 with lower values expected in the interstitial fluid around the bone cells [29]; however, the solubility of bone mineral increases dramatically when the pH drops below 7.0 [1,5]. Acidosis dramatically enhances the resorption activity of osteoclasts and, conversely, significantly inhibits the osteogenic function of osteoblasts, including the production of extracellular matrix, the activity of alkaline phosphatase and the formation of trabecular bone [4].

As citrate plays a pivotal role in the regulation of the acid–base equilibrium [9,30], the maintenance of its homeostasis is a basic requirement for this crucial function to be carried out. Citric acid is naturally contained in fruits and vegetables, particularly in citrus fruits, with concentration ranging from 0.005 mol/L in oranges and grapefruit to 0.30 mol/L in lemons and limes [31,32]. Almost the entire citrate intake is absorbed in the small intestine by means of a citrate transporter similar to that described in the kidneys [30,33]. Food citrate is rapidly introduced into the circulation, filtered at the glomerular level, and eventually reabsorbed according to physiological needs [30]. However, the net balance between gastrointestinal absorption and the urinary excretion of citrate suggests that the nutritional intake cannot be solely responsible for the maintenance of plasma homeostasis [13]. The citrate derived from the Krebs cycle marginally contributes to citrate homeostasis since it is used by cells as an energy source or for supporting specific cell functions [12]. It is currently well known that the main endogenous source of citrate is bone tissue, and the reasons for which so much citrate is found in bone have largely been clarified [6,13,34]. There is a link connecting citrate and bone as citrate (1) is produced by osteoblasts [12], (2) may influence their differentiation and functionality [19,20] and (3) serves to maintain the integrity of the skeletal nano- and microstructures [35]. In addition, all the conditions which upset the balance between bone formation and bone resorption may affect citrate homeostasis, including chronic low-grade acidosis [6]. On the one hand, a low pH stimulates osteoclast resorption and favours the mobilization of citrate stored in bone. Even though the unbound molecules could be used to form new mineral matrix, the main role of citrate in the case of acidosis is to maintain a constant citricemia and ensure renal excretion of the proton excess. On the other hand, the osteogenic function of bone-forming cells is inhibited, including the production of citrate as an essential component for the mineralisation of the extracellular matrix [4,5]. To translate the pathophysiology into clinical practice, the lower citrate bioavailability may lead to osteopenia or osteoporosis, i.e., a decrease in bone mass, deterioration of the skeletal microstructure, bone fragility and increased fracture risk [6]. In this regard, Chen et al. have demonstrated that serum citrate levels of elderly osteoporotic subjects were significantly lower than those of young healthy individuals and positively correlated with the bone mineral density of the lumbar spine and hip [36].

However, knowledge regarding the interaction between extracellular citrate and bone cells under acidic conditions is still lacking, and this gap does not allow identifying unequivocal indications for the use of citrate-based supplementation in the management of medical conditions related to acid overload and poor citrate bioavailability, and for mitigating its detrimental effect on skeletal homeostasis.

The exogenous supplementation of citrate may achieve two main functions: (1) citrate serves as an alkalizing agent because it is metabolised to 3 HCO^3−^ groups which, in turn, could act as a buffer and oppose the detrimental effects of the low pH and (2) the nutritional intake contributes to citrate homeostasis and may save the citrate released through the osteoclast resorption, thus making it reusable for the formation of new bone.

In the first step of the study, the effects of acidosis on the osteogenic properties of hMSCs, in particular on the citrate release and mineralisation of the extracellular matrix, were investigated. In the second step, we aimed to evaluate whether Ca citrate and K citrate, the citrate-based supplements most widely used in clinical practice, restored the impaired functions resulting from exposure to acidic conditions.

Other authors have shown that the detrimental effect of acidosis on bone cells was detectable at pH 6.9 [37], and therefore, this pH condition was chosen to mimic the acidic milieu for culturing and analysing the osteogenic properties of the hMSCs and the extracellular matrix organisation. The effects were explored at 14 days, in the early phase of matrix maturation and, then again at 21 days, thus providing adequate time for obtaining the formation of mineral nodules which could be microscopically identified using morphology and specific dyes [24]. The hMSCs used in the experimental plan were selected on the basis of their capability to form mineral nodules after 21 days of culture; their osteogenic commitment was also confirmed in large-scale gene expression profiling as reported in the Gene Expression Omnibus (GEO) dataset repository [38].

To the best of our knowledge, for the first time this study demonstrated that citrate excretion was significantly decreased when bone-forming cells lived in an acidic milieu, and that the decline was observed as early as 14 days. The above result could be explained in view of the knowledge that the mitochondrial aconitase and cytoplasmic citrate-lyase may increase during acidosis [39]; both enzymes favour the citrate consumption in the Krebs cycle and in fatty acid biosynthesis, respectively, thus reducing the release of citrate into the extracellular fluid. As a result, the net loss of the citrate bioavailability could have affected the mineralisation process. In fact, in the early phase, signs of a delay in matrix maturation were observed as the amount of collagen released in the supernatant and the initial deposition of calcium complexes into the extracellular matrix were significantly reduced. These findings did not depend on cell number and viability as these were similar at pH 6.9 and pH 7.4, thus suggesting that the effects induced by a low pH were related to the impairment of the cell function. Other authors have shown that the cell viability of osteogenic precursors was unaffected by the acidic condition if they were in a stationary phase of growth, corresponding to the confluence status of the hMSCs used in our experimental setting [37]. We verified that collagen released in the supernatant of the cells cultured at pH 6.9 did not derive from the degradation of the extracellular matrix, but was a newly synthesised collagen as the amount of Sirius Red staining correlated significantly with the procollagen type 1 N-terminal propeptide (P1NP). Osteoblasts make collagen in the form of procollagen which is excreted extracellularly, and the cleavage of N-terminal propeptide extensions, precedes the conversion of procollagen to mature collagen [40]. Even though the collagen release was lower, the amount of fibrils in the extracellular matrix was comparable to that measured at the neutral pH, thus suggesting that the acidic milieu did not hamper the collagen deposition. The literature data on the expression of type I collagen in acidic conditions are conflicting, but confirm the discrepancy between synthesis/release in culture medium and deposition into the extracellular matrix. In a previous study, we found that the expression of type I collagen was highly variable in acidic conditions but not significantly affected [18], while some authors showed that short-term exposure of mouse calvaria bone cells to acidic pH decreased the type I collagen mRNA [41], and others demonstrated that collagen deposition in acidic culture was augmented [37,42]. After 21 days of culture at pH 6.9, the quantitative differences in the collagen release and calcium deposition were no longer so evident, but the effects observed in the early phase compromised the final outcome as the number and size of the mineral nodules was reduced.

Areas in which the mineralisation nodules were not clearly visible were also evaluated by using TEM and FT-IR spectroscopy, with the aim of highlighting the submicroscopic changes in the organisation and composition of the matrix. Under the acidic conditions, the ultrastructural TEM images showed an impaired arrangement of the collagen and the lack of matrix vesicles aligned to the fibres. Fibrillar collagen provides a template for the mineral deposition since spindle- or plate-shaped crystals of hydroxyapatite tend to be oriented in the same direction as the fibres [43]. Mutations in the aminoacid sequence of collagen, defects as disorganised collagen fibres, as well as pathological accumulation of unfolded collagen triple helices, impair fibrillar collagen functions and, ultimately, tissue mineralisation [44]. In human cells, the damage seemed to be more evident than that observed in the murine model as the osteoblast cultures derived from rat calvaria did not exhibit notable differences in the organisation of collagen fibrils at pH 6.9 and pH 7.4 [37].

FT-IR spectroscopy has been proposed as a powerful technique for the characterisation of proteins and collagen-based materials, and we focused on Amide I and Amide II which are also considered to be the two main markers of collagen structure [45,46,47,48,49]. Differences in the intensity of the Amide II band suggested that collagen orientation and organisation could be affected by pH and supported the TEM observation of a disorder in the fibril arrangement [45].

To assess the presence of mineralisation nuclei in areas where mineral nodules were not microscopically visible was a challenging and scarcely investigated application in the field of FT-IR spectroscopy. We were expecting to find a low amount of mineralised phases and close to the detectability threshold of the instrument. Furthermore, the heterogeneity of cell culture components, i.e., cells, matrix and culture medium, generated several bands through the spectrum which tended to conceal those object of study. Nevertheless, we were able to identify the phases of interest and to highlight the qualitative differences among the curves related to the culture conditions [27,28]. The FT-IR analysis demonstrated that the bands corresponding to calcium phosphates were detectable even when the mineral nodules were not microscopically visible, thus suggesting that submicrometric mineralisation was beginning [50,51,52,53]. In agreement with the result obtained by measuring the amount of Alizarin Red bound to the calcium complexes, the extent of the mineralisation did not change at pH 6.9, but the the marked presence of the octacalcium-phosphate phase was consistent with a deviation of the mineralisation process. Studies regarding mineral formation have demonstrated that the least soluble calcium-phosphate phase, hydroxyapatite, was preferentially formed under neutral or basic conditions while a low pH favoured the less stable phases, i.e., octacalcium phosphate which is a precursor of bone apatite formation [54]. Our findings showed that FT-IR analysis provided relevant information by qualitative comparison of the curves. Based on these encouraging results, we could further exploit the FT-IR spectroscopy to determine in a larger number of samples the exact number of phases formed and to evaluate the differences among samples statistically.

To better support the results observed with biochemical, morphological and ultrastructural analyses, we also evaluated the expression of two genes that play an essential role in mineralization of the extracellular matrix [55]. Osteonectin/SPARC is a secreted protein acidic and rich in cysteine that is required for the regulation of procollagen processing and assembly in the bone matrix, mineral incorporation and cross-linking. There is a close association between SPARC and collagen I expression, and its capacity to bind to collagen is a critical step of the mineralization process. Indeed, SPARC-null osteoblasts show similar levels of osteoblast differentiation markers, including bone sialoprotein, but the formation of mineralized nodules is impaired [56]. Bone sialoprotein/IBSP belongs to the “small integrin-binding ligand N-linked glycoproteins” (SIBLING) family, which is an extracellular matrix protein family playing a critical role in the mineralisation process. Bone sialoprotein binds to calcium, induces nucleation of hydroxyapatite crystals in vitro, and is crucial for the structure of the mineralised matrix. Furthermore, IBSP can bind to collagen fibrils, especially to their hole zones that are the site of early mineral deposition [57].

The acidic milieu determined a reduction in osteonectin/SPARC expression, thus providing a molecular basis for a possible explanation of the observed events, i.e., the decrease in collagen release, the ultrastructural disorganization in the extracellular matrix, the prevalence of precursor of bone apatite, and the reduced formation of mineralization nodules. Conversely, IBSP transcription was slightly increased, but the upregulation, while ensuring the precipitation of amorphous calcium-phosphate, was not sufficient to compensate for the altered phenotype, probably due to the excessive disorganization of the extracellular matrix.

On the basis of the above results, it was reasonable to hypothesise that the decreased citrate release induced by the acidic microenvironment could have been involved in a decreased ability for mineralisation. The role of citrate in driving the mineralisation process has been well recognised as it is an integral part of the apatite-collagen nanocomposite and contributes to controling the size, longitudinal growth and thickness of the apatite nanocrystals in achieving the typical plate-like morphology which ensures the biomechanical properties of bone, including stability, strength, and resistance to fracture [58,59,60]. That the “osteoblast citration” is a fundamental step of bone formation has been conceptualised by Costello et al. (2012) who argued that “mineralization without citration will not result in the formation of normal bone, i.e., bone that exhibits its important properties, such as stability, strength, and resistance to fracture” [61].

The aim of the next step of the study was to demonstrate that the impairment of the mineralisation process due to an acidic milieu could have been opposed by supplying the missing citrate, thus resulting in the same amount released by the hMSCs cultured under neutral conditions. For this purpose, the citrate supplementation used in the present experimental plan took into account the real deficit found under acidic conditions. Two different citrate sources were evaluated, namely Ca citrate and K citrate, which are also the citrate-based compounds commonly used in clinical practice.

When comparing the effect of the citrate supplementation under acidic conditions with what was observed under neutral conditions, the result could be considered satisfactory where significant differences were no longer observed, thus responding to the objective.

Both compounds exhibited alkalizing properties, but they were not able to fully restore the neutral pH as all the concentrations used raised the pH value of the culture medium to over 7.0 but the maximum increase, pH 7.27, was observed only with the highest dose of K citrate. Nevertheless, the functions for the most part impaired under the acidic conditions, i.e., collagen release and mineralisation, were partially or fully recovered after citrate supplementation, having different effects according to the compound used.

Overall, the intermediate doses were the most effective while the highest concentration, which was non-cytotoxic in the short-term preliminary test, showed an inhibitory effect on some parameters in the long term, and it cannot be excluded that this was due to the excess of Ca^2+^ or K^+^ in the culture medium. Ca^2+^ or K^+^ may influence not only the ion balance and the extracellular pH but also influence multiple cellular functions, including molecular pathways, ion channels and membrane transporter. Regarding potential cytotoxic effects, in different experimental settings, other authors demonstrated that elevated concentrations of extracellular Ca^2+^, until 5 mM, promoted cell viability and late-stage osteogenic differentiation of MSCs, but may suppress early-stage osteogenic differentiation [62]. A recent study of Gao et al. (2018) showed that MSCs could endure K^+^ concentrations ranging from 5 to 130 mM, but high concentrations may impair proliferation and induce apoptosis [63]. In the above studies, Ca^2+^ and K^+^ concentrations were much higher than those employed in our experimental plan.

In the first phase of the culture, the supplementation was not able to correct the notable decrease in collagen release which was found under the acidic conditions. However, after 21 days, both compounds increased the collagen bioavailability, even more than in the control cultures, thus suggesting that the positive effect was ascribable to citrate irrespective of the source, although the presence of calcium induced a faster response.

Instead, the first 14 days were sufficient for the recovery of mineralisation and to make it similar to that observed under the neutral conditions. The Ca citrate was slightly more effective than the K citrate, but the difference became more evident in the late phase. In fact, at 21 days, the Ca citrate showed notable pro-osteogenic activity and, in spite of the unfavourable microenvironment, fostered the mineralisation process even exceeding what was observed at pH 7.4 while the initial positive effect exhibited by the K citrate seemed to be exhausted. This result confirmed what we demonstrated in previous studies in which was that, although it showed significant anti-osteoclastogenic activity, the K citrate was not as effective in promoting mineralisation [18]. We cannot exclude that the decreased mineralization ability observed with the highest concentration of citrate-based compounds under acidic condition could depend on the decalcifying properties of citric acid. The capability to dissolve calcium compounds is also exploited for medical purposes, especially in dentistry, but the decalcifying properties strongly depend on pH value and concentration, usually lower than pH 6.9 and higher than 0.56 mg/mL, respectively [64]. Both the above conditions differ significantly from those applied in our experimental setting.

While the release of collagen into the culture supernatant increased after citrate supplementation, the amount deposited in the extracellular matrix tended to decrease; however, this reduction did not correlate with the ability to accomplish the mineralisation process. Rather, the mineralisation observed at the final time point faithfully reflected the ability to release the collagen which was observed in the early phase of the culture, thus suggesting a sequential connection of events: the better the collagen bioavailability at 14 days, the better the mineralisation capability observed at 21 days; conversely, the higher the extent of the mineralisation observed at 21 days, the lower the need of new collagen deposition into the extracellular matrix. After treatment with citrate-based supplements, the expression osteonectin/SPARC and IBSP expression was highly variable and no significant changes were detectable.

Positive effects on the collagen release and mineralisation were also observed under neutral conditions, thus testifying that citrate concentrations exceeding what is available in the physiological microenvironment do not hamper the osteogenic potential of bone-forming cells. However, Ca citrate seemed more effective than K citrate in promoting the mineralisation, also favouring the collagen release and the IBSP expression. Additional evidence of the pro-osteogenic effect of the citrate supplementation emerged from the spectroscopy analysis which revealed that the extent of the submicrometric mineralisation activity in areas lacking in large mineral nodules was promoted by both supplements. This was correlated with the citrate concentration rather than with the type of compound, although in this setting the K citrate exhibited a slightly better effect than the Ca citrate. We had previously found that K citrate did not have a considerable osteopromotive activity and these results were herein confirmed [18]. However, by making an in-depth study of the extracellular matrix, some aspects which had been undetected earlier were highlighted.

This study enhances the knowledge regarding the pro-osteogenic activity of citrate supplements in an acidic microenvironment, while previously published data were obtained mainly under neutral conditions. Costello et al. (2015) have demonstrated that the BMP2 induction of osteoblast differentiation and mineralisation was related to the citrate released from osteogenic precursors [19]. Ma et al. (2018) have shown that extracellular citrate fostered a “metabonegenic” regulation of intracellular events in preparation for the osteogenic differentiation of the hMSCs. The citrate uptake affected downstream osteophenotype progression and favoured the metabolic switch from glycolysis to oxidative respiration to generate more ATP and meet the high energy demands required for the production of matrix proteins [20].

For many years now, citrate-based supplements have been proposed in the clinical setting for treating patients affected by disorders of bone-remodelling, such as osteopenia and osteoporosis. In particular, Ca citrate is used when calcium supplementation is required for preventing bone loss [65], and K citrate has been proposed as a strategy capable of opposing the acid overload and the deleterious effects on bone health status [66]. However, the numerous clinical trials did not consider in their rationale that citrate, by itself, plays an essential role in maintaining bone health, as proved by the previously cited experimental studies and also supported by clinical evidence. Low citrate excretion has been found in a considerable proportion of osteopenic women [16] and there is a strong relationship between urinary citrate excretion and the prevalence of fragility fracture in postmenopausal women [30]; plasma citrate levels correlate with the bone mineral density of the lumbar spine and hip [36]. By comparing the results obtained in this study with the results of interventional clinical trials, a “common ground” emerges. Overall, in the clinical setting, an adequate calcium intake is fundamental for preventing bone loss; however, Ca citrate seems to be more effective than calcium carbonate [67,68,69]. Potassium citrate limits bone loss in postmenopausal women and elderly subjects, with or without osteoporosis [70,71,72,73,74,75] and also anabolic effects with increased circulating levels of bone formation markers, i.e., PINP, have been reported [16,74]; combined treatment K citrate and Ca citrate allows obtaining an additional decrease in bone turnover [76]. Finally, positive effects of citrate supplementation have also been observed in the absence of an excessive acid load [77].

## 5. Conclusions

The present results proved that the acidic milieu negatively affected an essential requirement for obtaining effective mineralisation, i.e., the citrate release from osteogenic precursors. By means of supplying the missing quantity using citrate-based supplements, the impaired mineralisation capability may be restored. Citrate, by itself, improves some functions, such as collagen release and the beginning of mineralisation with deposition of submicrometric calcium complexes into the extracellular matrix. However, according to what has been previously observed in interventional trials, the mineralisation process seems to be more effective when Ca citrate is used. These findings provided a biological basis to support the use of citrate-based supplements in the management of bone-remodelling disorders, especially when there is low-grade acidosis and poor citrate availability. The experimental model allowed investigating the biological effects of these compounds by mimicking the altered microenvironment, however, in a simplified setting which eliminated the complex influences of other pathophysiological mechanisms which were not reproducible in vitro. Therefore, extreme caution must be used when interpreting the data from such an approach; the clinical judgement of practitioners is essential in identifying patients who could reap real benefits and the conditions under which the supplementation should be avoided. The search for signs of a chronic low-grade acidosis and alteration of the citrate homeostasis should be included in the diagnostic workup of patients affected by metabolic bone diseases. Identifying patients who exhibit the target conditions and could take advantages from the dietary supplementation is a starting point for further clinical trials aimed to recognise the benefits deriving from citrate-based compounds.

## Supplementary Materials

The following are available online at https://www.mdpi.com/2072-6643/12/12/3779/s1, Figure S1: The effects of acidic milieu and citrate supplementation on the expression of SPARC and IBSP of hMSCs after 14 days of treatment. Results are expressed as ratio between the expression of “gene of interest” and “YWHAZ” as housekeeping gene. The bars represent the mean ± SEM of two experiments. (a) SPARC expression and (b) IBSP expression under neutral and acidic conditions; (c) SPARC and (d) IBSP expression after treatment with citrate-based supplements under acidic conditions; (e) SPARC and (f) IBSP expression under neutral condition. * *p* < 0.05 pH 7.4 vs. pH 6.9; § *p* < 0.05 vs. 0 mg/mL citrate supplements; *p* < 0.05 Ca citrate vs K citrate, Figure S2: Results of FT-IR microscopy analysis. The upper panel shows the imaging of samples at (a) pH 7.4, (b) pH 6.9 and of (c) MSCs. In the lower panel, FT-IR spectra in the 700–1200 cm^−1^ region, as obtained by FT-IR microscopy analysis of hMSC monolayers. (d) Curves of non-mineralising MSCs (reference curve for FT-IR analysis); (e) curves of hMSCs cultured in osteogenic medium in neutral condition (pH 7.4); (f) curves of hMSCs cultured in osteogenic medium in acidic condition (pH 6.9). Spectra show different profiles and degrees of internal variability. The arrows indicate the area of the bands in the 1000–1100 cm^−1^ region, characteristic of phosphates stretching and hence, indicative of mineralisation, Figure S3: Evaluation of alkalising capability and cytotoxicity of citrate-based compounds. (a) The upper panel shows that the supplementation with citrate-based compounds was not sufficient to completely neutralise the acidic medium. No toxic effect was observed at the tested concentration for Ca Citrate supplement both in neutral (b) and acidic (c) conditions. An increase in cell viability was observed with low and medium doses of Ca citrate in neutral and acidic concentration, respectively. No toxic effect was observed at the tested concentration for K Citrate supplement both in neutral (d) and acidic (e) conditions, Figure S4: Effect of Ca citrate and K citrate on mineralisation (FT-IR analysis). Curves are reported after subtracting the reference in absence of citrate supplementation (Ctrl pH 7.4). A relevant difference is visible in the extent of mineralisation depending on the citrate concentration and, for higher concentrations, on the supplement type. The shift in the alignment of the phosphates stretching bands suggests differences in calcium phosphates phase formation.
