# Supplementary material for: Citrate Supplementation Restores the Impaired Mineralisation Resulting from the Acidic Microenvironment: An In Vitro Study
Source: Nutrients. 2020 Dec 9;12(12):3779. doi: 10.3390/nu12123779 (PMC7763163; doi:10.3390/nu12123779)
Supplement: Supplementary file 1 [file nutrients-12-03779-s001.pdf]

nutrients

MDPI

Supplementary materials

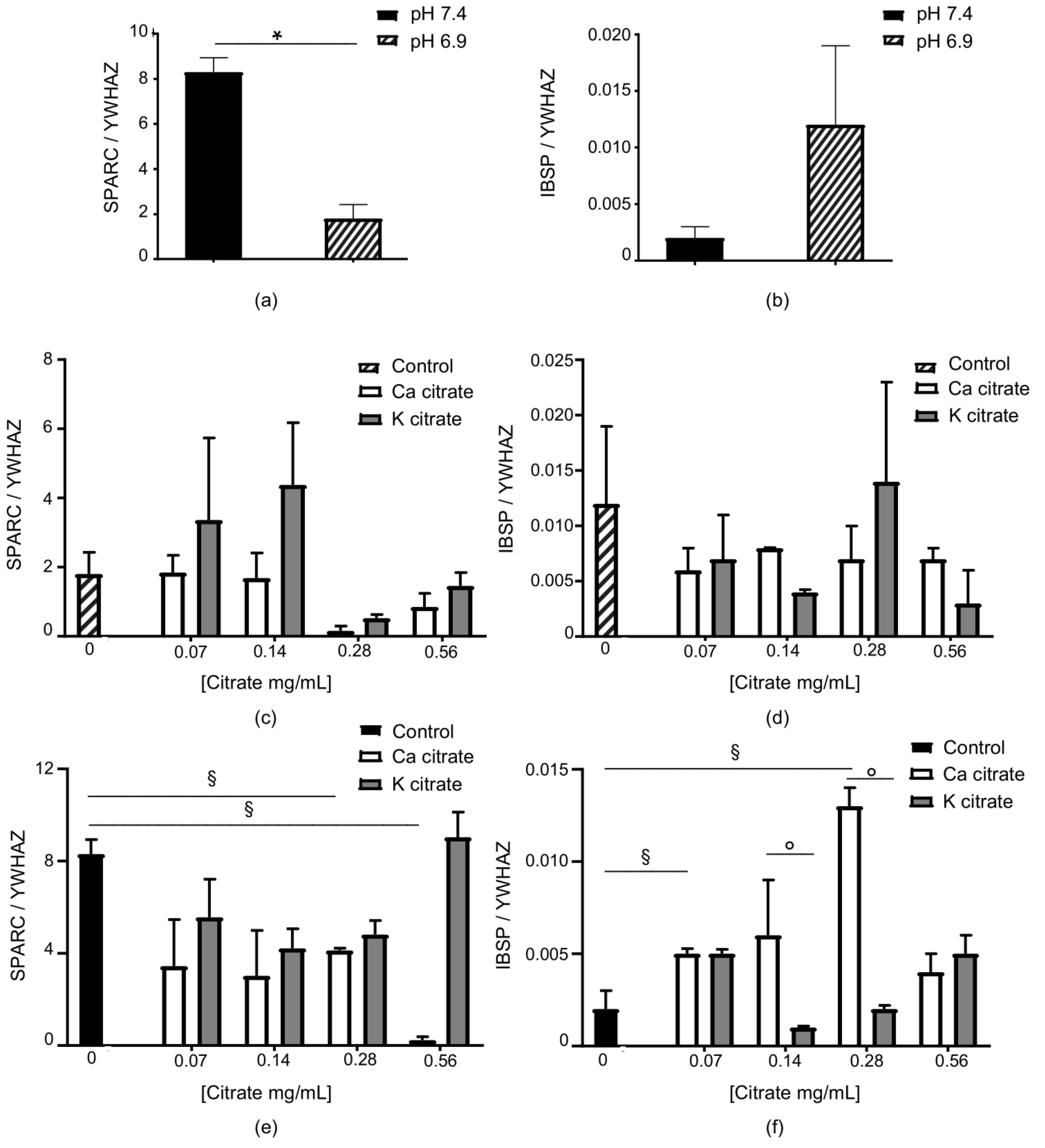

Figure S1. The effects of acidic milieu and citrate supplementation on the expression of SPARC and IBSP of hMSCs after 14 days of treatment. Results are expressed as ratio between the expression of “gene of interest” and “YWHAZ” as housekeeping gene. The bars represent the mean ± SEM of two experiments. (a) SPARC expression and (b) IBSP expression under neutral and acidic conditions; (c) SPARC and (d) IBSP expression after treatment with citrate-based supplements under acidic conditions; (e) SPARC and (f) IBSP expression under neutral condition. * $P < 0.05$ pH 7.4 vs pH 6.9; § $P < 0.05$ vs 0 mg/mL citrate supplements; ° $P < 0.05$ Ca citrate vs K citrate.

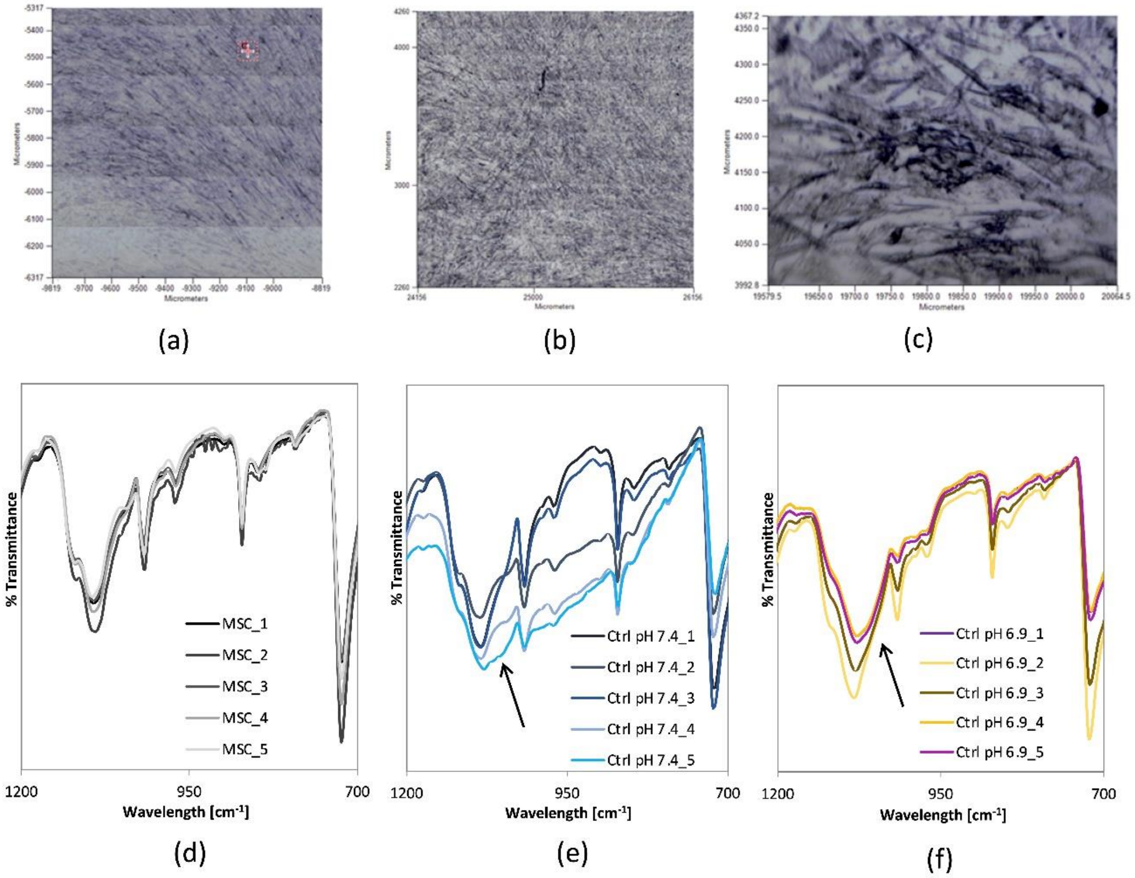

Figure S2. Results of FT-IR microscopy analysis. The upper panel shows the imaging of samples at **(a)** pH 7.4, **(b)** pH 6.9 and of **(c)** MSCs. In the lower panel, FT-IR spectra in the 700-1200 cm^{-1} region, as obtained by FT-IR microscopy analysis of hMSC monolayers. **(d)** Curves of non-mineralising MSCs (reference curve for FT-IR analysis); **(e)** curves of hMSCs cultured in osteogenic medium in neutral condition (pH 7.4); (f) curves of hMSCs cultured in osteogenic medium in acidic condition (pH 6.9). Spectra show different profiles and degrees of internal variability. The arrows indicate the area of the bands in the 1000-1100 cm^{-1} region, characteristic of phosphates stretching and hence, indicative of mineralisation.

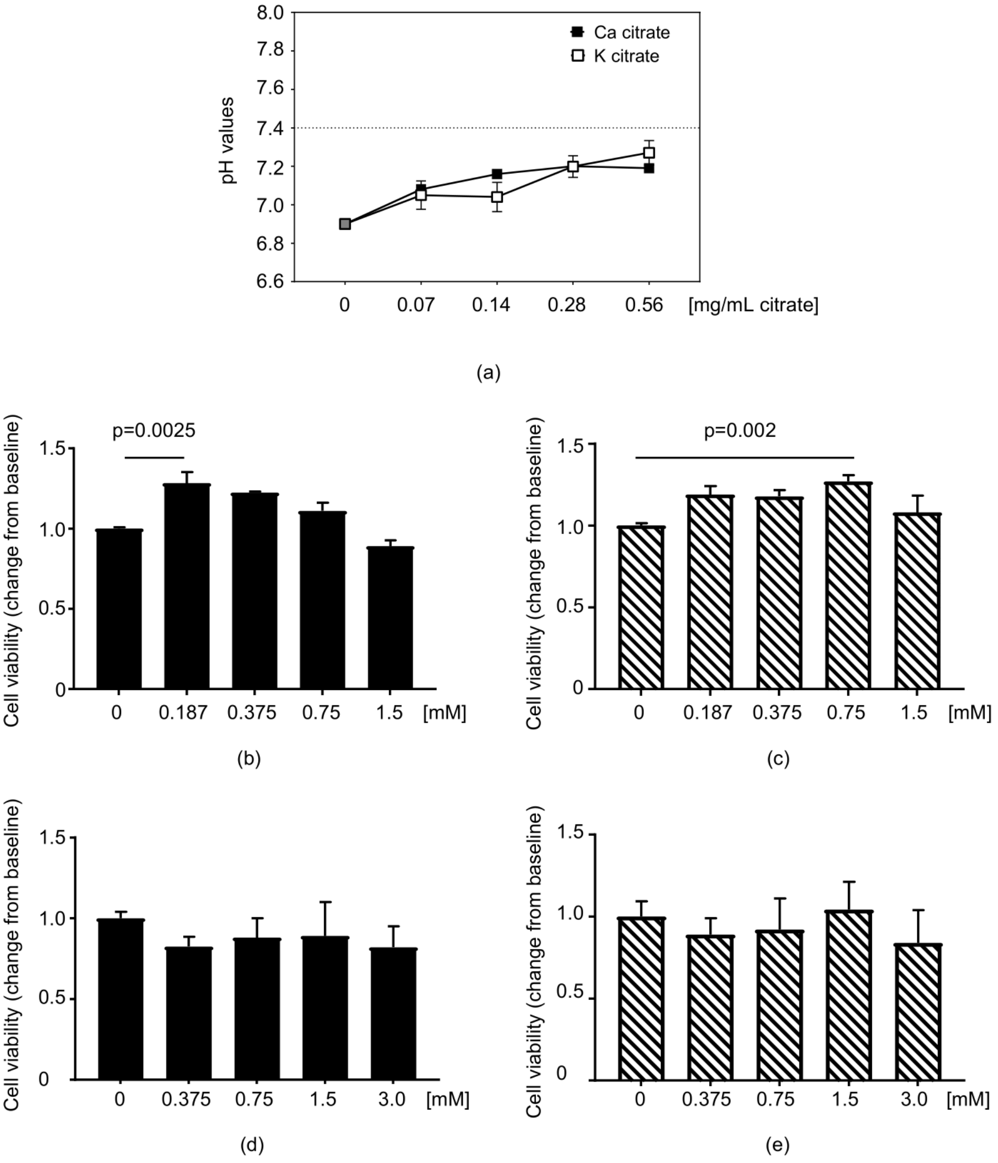

Figure S3. Evaluation of alkalising capability and cytotoxicity of citrate-based compounds. **(a)** The upper panel shows that the supplementation with citrate-based compounds was not sufficient to completely neutralise the acidic medium. No toxic effect was observed at the tested concentration for Ca Citrate supplement both in neutral **(b)** and acidic **(c)** conditions. An increase in cell viability was observed with low and medium doses of Ca citrate in neutral and acidic concentration, respectively. No toxic effect was observed at the tested concentration for K Citrate supplement both in neutral **(d)** and acidic **(e)** conditions.

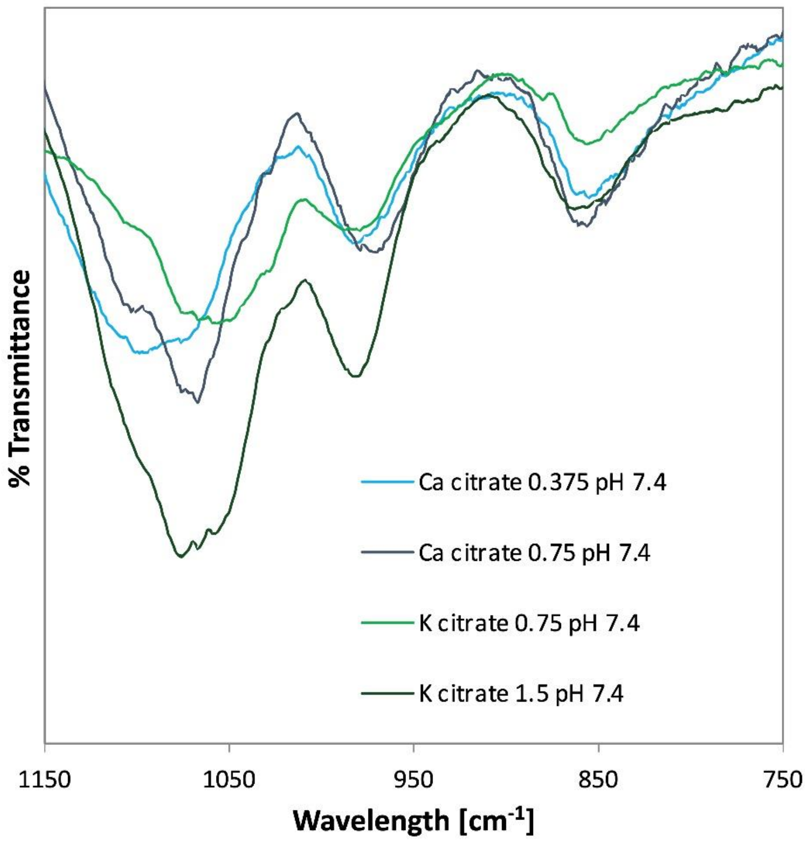

Figure S4. Effect of Ca citrate and K citrate on mineralisation (FT-IR analysis). Curves are reported after subtracting the reference in absence of citrate supplementation (Ctrl pH 7.4). A relevant difference is visible in the extent of mineralisation depending on the citrate concentration and, for higher concentrations, on the supplement type. The shift in the alignment of the phosphates stretching bands suggests differences in calcium phosphates phase formation.
